# Plasma levels of matrix metalloproteinase-9 are elevated in individuals with hypertensive crisis

**DOI:** 10.1186/s12872-020-01412-5

**Published:** 2020-03-12

**Authors:** Flavia Mariana Valente, Days Oliveira de Andrade, Luciana Neves Cosenso-Martin, Cláudia Bernardi Cesarino, Sérgio Mussi Guimarães, Victor Beneditti Guimarães, Riccardo Lacchini, José Eduardo Tanus-Santos, Juan Carlos Yugar-Toledo, José Fernando Vilela-Martin

**Affiliations:** 1 Internal Medicine Department, Hypertension Clinic, State Medical School at Sao Jose do Rio Preto (FAMERP), Ave Brig Faria Lima, 5416, Sao Jose do Rio Preto, SP 15090-000 Brazil; 2 Medical School FACERES, Sao Jose do Rio Preto, SP Brazil; 3 Department of Psychiatric Nursing and Human Sciences, Ribeirao Preto College of Nursing, University of Sao Paulo, R. Prof. Helio Lourenço, Ribeirao Preto, SP 3900 Brazil; 4 Department of Pharmacology, Ribeirao Preto Medical School, University of Sao Paulo, Ave Bandeirantes, Ribeirao Preto, SP 3900 Brazil

**Keywords:** Matrix metalloproteinase, Cardiac biomarkers, Hypertension, Emergency, Cardiovascular pathophysiology

## Abstract

**Background:**

Matrix metalloproteinase-9 (MMP-9) participates in the degradation of components of the extracellular matrix and it is involved in vascular remodeling and vasomotor changes. The aim of this study was to investigate the plasma levels of MMP-9 in acute vascular alterations due to hypertensive crisis.

**Methods:**

This cross-sectional study was performed in 40 normotensive (NT) and 58 controlled hypertensive subjects (CHyp) followed up in outpatient clinic. Moreover, 57 patients with hypertensive emergency (HypEmerg) and 43 in hypertensive urgency (HypUrg), seen in emergency department, were also included. Hypertensive crisis was divided into HypEmerg, which was characterized by levels of systolic blood pressure (BP) ≥ 180 mmHg and/or diastolic BP ≥ 120 mmHg complicated with target-organ damage (TOD), and HypUrg, defined by BP elevation without TOD. Univariate and multivariate regression analysis was performed to identify the influence of independent variables on MMP-9 levels. A *p*-value < 0.05 was considered statistically significant.

**Results:**

The mean age was 43.5 years in the NT group (11 men); 57.7 years in the CHyp group (29 men); 59.4 years in the HypUrg group (21 men) and 62.4 years in the HypEmerg group (31 men). The age was statistically different in the NT group compared to other 3 groups. The mean BP was 116.5 ± 13.9/72.4 ± 10.6 mmHg for NT, 123.2 ± 12.6/79 ± 9.2 for CHyp, 194.1 ± 24.3/121.4 ± 17.3 for HypUrg and 191.6 ± 34.3/121.7 ± 18.8 mmHg for HypEmerg, respectively (*p*-value< 0.0001 between groups). MMP-9 levels were statistically different between the HypEmerg (2.31 ± 0.2 ng/mL) and HypUrg groups (2.17 ± 0.3 ng/mL) compared to the NT (1.94 ± 0.3 ng/mL) (*p-value* < 0.01 and *p-value* < 0.05, respectively) and CHyp groups (1.92 ± 0.2 ng/mL) (*p-value* < 0.01). Uric acid was the only independent variable for predicting MMP-9 levels (*p*-value = 0.001).

**Conclusion:**

MMP-9 concentrations are significantly higher in the hypertensive crisis groups (urgency and emergency) compared to the control groups. Therefore, MMP-9 may be a biomarker or mediator of pathophysiologic pathways in cases of acute elevations of blood pressure.

## Background

Systemic arterial hypertension is an important public health problem and a modifiable risk factor closely associated with cardiovascular disease (CVD), the leading cause of death worldwide [1]. In this context, clinical trials have shown that the reduction in blood pressure (BP) levels results in a reduction in the risk of developing strokes, coronary artery disease, heart failure and renal insufficiency [2].

Vascular remodeling, an adaptive response that attempts to restore the vascular mechanical balance and the BP, is one of several pathophysiological mechanisms that characterize the multifactorial etiology of hypertension [3, 4]. However, pathological remodeling is associated with vascular changes including endothelial dysfunction, smooth muscle cell hypertrophy of the arteries, and cell migration and proliferation with resulting thickening of the vascular wall and structural changes of the extracellular matrix (ECM) [5, 6].

Degradation and reorganization of the vascular wall matrix results from the activation of proteolytic pathways such as matrix metalloproteinases, especially gelatinase (MMP-2 and MMP-9), a group of zinc-dependent endopeptidases whose imbalance between activation and inhibition results in excessive degradation of ECM proteins [6–8].

Recently, high levels and activity of MMP-2 and MMP-9 have been demonstrated in hypertensive patients [9, 10] and in animal models [5, 7, 11, 12]. The levels of MMP-9 have been associated with cardiovascular diseases due to its proteolytic activity on type IV collagen, one of the main constituents of the basal membrane that involves vascular smooth muscle cells and the endothelium, exerting an important role in cell migration and infiltration in the atherosclerotic process [13]. In addition, the degradation of elastin by MMP-9 is implicated in the process of arterial stiffness and the development of aneurysms [8, 10, 14].

In view of the scarcity of studies evaluating MMP-9 levels at different BP levels and especially with acute and marked elevations of BP, the objective of the present study was to investigate MMP-9 as a biological pathway and/or biomarker of acute vascular alterations resulting from severe BP elevations characterized as hypertensive crisis.

## Methods

### Enrollment of patients and sample collection

The Research Ethics Committee of the institution approved the study protocol according to national and international guidelines (CAAE no. 07606212.5.0000.5415, no. 94.248/2012). Subsequently, the individuals were informed about the objectives of this study and consulted about their interest and consent to participate as volunteers, in a way that, regardless of their choice, their treatment would not suffer. The current study was performed according to the ethical standards of the Helsinki Declaration.

The control group consisted of 40 normotensive patients (NT) who had systolic blood pressure (SBP) < 140 mmHg and diastolic blood pressure (DBP) < 90 mmHg without taking antihypertensive drugs and they were followed up in outpatient clinic for other reasons not related to hypertension. The controlled hypertensive group (CHyp) comprised 58 subjects with SBP < 140 mmHg and DBP < 90 mmHg while taking antihypertensive drugs, who were being followed up in a university service specialized in hypertension and agreed to participate in the study. Thus, normotensive patients were selected from other specialties in the outpatient service of the university hospital and the controlled hypertensive individuals were followed up in hypertension service (hypertension clinic) of the same hospital.

A total of 100 individuals aged ≥18 years presenting hypertensive crisis and subsequently admitted to the Clinical Emergency Department of the university hospital were evaluated in the third group (patients lived in the city, specifically in the Hospital area). They were grouped as hypertensive emergency (*n* = 57) and urgency patients (*n* = 43). The hypertensive emergency group (HypEmerg) was characterized by subjects with elevated levels of SBP ≥ 180 mmHg and/or DBP ≥ 120 mmHg complicated by evidence of progressive acute target organ damage (TOD), such as hypertensive encephalopathy, hemorrhagic or ischemic stroke, acute myocardial infarction, left ventricular failure with acute pulmonary edema, unstable angina pectoris, acute aortic dissection or acute and progressive renal failure. On the other hand, hypertensive urgencies (HypUrg) were defined as increases in BP without TOD [1, 15].

The exclusion criteria for all the groups adopted included previous diagnosis of hypertension or prior use of antihypertensive drugs (valid for normotensive participants), chronic diseases that could limit participation in the study (e.g. tumors), difficulty in understanding, inability to measure BP, and refusal to sign the informed consent form. Additionally, female patients presenting with preeclampsia and eclampsia, and hypertensive patients with pseudocrisis were excluded. Emergency Department BP was measured in triplicate according to VII National Joint Committee guidelines [15], using an automatic digital blood pressure monitor (OMRON Healthcare Inc., Bannockburn, IL, USA). The recorded BP was the mean of the three readings.

All patients aged 18 years and above who presented with hypertensive emergency and hypertensive urgency were included in the study. The same age criterion was used for normotensive and controlled hypertensive groups. An investigative protocol was used to collect information on patient history, associated diseases (diabetes mellitus), medications, smoking, and family history. The weight and height were measured using anthropometric scales.

### Biochemical evaluation

Venous blood was collected to measure serum glucose, total cholesterol (TC), high-density lipoprotein cholesterol (HDL-c), triglycerides (TG), creatinine, potassium, and uric acid levels. The LDL-c fraction was calculated for TG < 400 mg/dL using the formula LDL-c = TC - HDL-c - TG/5. The glomerular filtration rate (GFR) was calculated by the Chronic Kidney Disease Epidemiology Collaboration - CKD-EPI creatinine equation. Citrated venous blood for ELISA was also collected in EDTA vacutainer tubes (Becton-Dickinson, São Paulo, Brazil) by venipuncture, and centrifuged at 3500 rpm for 10 min with plasma fractions being immediately stored at − 70 °C until measurement of the MMP-9.

### Enzyme immunoassays of plasma MMP-9

The levels of MMP-9 were measured by a commercially available sandwich enzyme-linked immunosorbent assay kit (DY911; R&D Systems, Minneapolis, MN, USA), according to the manufacturer’s instructions.

### Statistical analysis

Data are presented as means ± standard deviation (SD) for continuous variables and as percentages (%) for categorical variables. The comparison between the groups in relation to the continuous variables was performed by analysis of variance (ANOVA) for parametric and Kruskal-Wallis for non-parametric test. Qualitative variables were analyzed using the chi-square or Fisher’s exact test. The results of MMP-9 are presented with a calculation of medians (ng/mL). Subsequently, MMP-9 values were transformed into logarithms to reflect normal distribution for statistical analysis. Univariate and multiple linear regression analysis were performed to identify the influence of independent variables on logMMP-9 levels. The variables that presented *p*-values < 0.05 in the univariate analysis were included for posterior multiple linear regression analysis. A *p*-value < 0.05 was considered statistically significant.

## Results

The study included individuals aged between 22 and 92 years old with a mean age of 43.5 years in the NT group (11 men); 57.7 years in the CHyp group (29 men); 59.4 years in the HypUrg group (21 men) and 62.4 years (31 men) in the HypEmerg group. Table 1 shows the characteristics of the population of the present study including BP levels and medications taken, and Table 2 shows the levels of the analyzed biochemical variables.

**Table 1:** Clinical characteristics of normotensive subjects and controlled hypertensive patients and those in hypertensive crisis (divided in hypertensive urgency and emergency)

Variable	NT (*n* = 40)^a^	CHyp (*n* = 58)^b^	HypUrg (*n =* 43)^c^	HypEmerg (*n* = 57)^d^	*p-*value *(axbxcxd)*	a x b	a x c	a x d	b x c	b x d	c x d
Age (years)	43.5 ± 10.2	57.7 ± 7.4	59.4 ± 15.6	62.4 ± 14.3	**<0.0001**	**<0.01**	**<0.01**	**<0.01**	0,09	0.10	0.09
Gender (male; %)	11 (27.5)	29 (50)	21 (48.9)	31 (54.4)		**0.03**	0.16	**0.01**	0.20	0,24	0.16
Skin color (White; %)	33 (82.5)	50 (86.2)	27 (62.8)	44 (77.2)		0.12	0.10	0.12	**0.009**	0.11	0.12
BMI (kg/m^2^)	23.7 ± 3.3	29.6 ± 3.4	30.1 ± 7.2	27.6 ± 6.3	**0.002**	**<0.01**	**<0.01**	**0.006**	0.17	**0.01**	**0.03**
Smokers (%)	6 (15%)	11 (19)	7 (16.3)	17 (29.8)		0.15	0.14	0.14	0.09	0.10	0.08
History of diabetes (%)	–	9 (15.5)	12 (27.9)	23 (40.3)		**0.009**	**0.0002**	**0.0001**	0.14	**0.003**	0.09
***Blood Pressure***											
SBP (mmHg)	116.3 ± 11.8	123.2 ± 10.5	194.1 ± 28.6	191.6 ± 34.3	**<0.0001**	0.16	**<0.01**	**<0.01**	**<0.01**	**<0.01**	0.10
DBP (mmHg)	72.3 ± 9	79 ± 7.7	121.4 ± 17.3	121.7 ± 18.8	**<0.0001**	0.14	**<0.01**	**<0.01**	**<0.01**	**<0.01**	0.09
***Drugs, n*(%)**											
Antidiabetic drugs (%)	–	16 (27.6)	4 (9.3)	13 (22.8)		–	–	–	**0.02**	0.12	0.11
Statins (%)	–	14 (24.1)	15 (34.9)	22 (38.6)		–	–	–	0.14	0.20	0.14
Diuretics (%)	–	48 (82.8)	26 (60.4)	29 (50.9)		–	–	–	**0.02**	**0.0003**	0.12
ARB (%)	–	25 (43.1)	16 (37.2)	18 (28)		–	–	–	0.09	0.14	0.17
ACEi (%)	–	22 (38)	18 (41.8)	31 (45.6)		–	–	–	0.17	0.18	0.14
CCB (%)	–	18 (31)	14 (32.6)	18 (31.6)		–	–	–	0.14	0.20	0.22
B-blockers (%)	–	16 (27.6)	26 (60.4)	27 (47.4)		–	–	–	**0.001**	**0.03**	0.18
Antiaggregant and/or anticoagulant (%)	–	19 (32.8)	19 (44.2)	37 (64.9)		–	–	–	0.20	**0.0008**	**0.04**

*NT* normotensive group, *CHyp* controlled hypertensive group, *HypUrg* hypertensive urgency, *HypEmerg* hypertensive emergency, *BMI* body mass index, *SBP* systolic blood pressure, *DBP* diastolic blood pressure, *ARB* angiotensin receptor blocker, *ACEi* angiotensin converting enzyme inhibitor, *CCB* calcium channel blockers

**Table 2:** Biochemical parameters of normotensive subjects and controlled hypertensive patients and those in hypertensive crisis (divided in hypertensive urgency and emergency)

Variable	NT (***n*** = 40)^**a**^	CHyp (***n*** = 58)^**b**^	HypUrg (***n =*** 43)^**c**^	HypEmerg (***n*** = 57)^**d**^	***p*** value ***(axbxcxd)***	a x b	a x c	a x d	b x c	b x d	c x d
***Biochemical parameters***											
Glycemia (mg/dL)	86.6 ± 16.7	104.8 ± 42.6	121.5 ± 45.5	149.7 ± 96.3	**<0.0001**	**0.007**	**0.002**	**0.003**	**0.04**	**0.04**	0.66
HDL-c (mg/dL)	61.2 ± 12.2	53 ± 13.2	52.6 ± 19.8	48.1 ± 16.4	**0.008**	**0.02**	**0.004**	**0.006**	**0.001**	**0.002**	**0.004**
LDL-c (mg/dL)	115.2 ± 23.3	123.6 ± 31.8	127.1 ± 38.5	109.3 ± 44.8	**0.03**	0.09	0.18	0.08	0.84	0.70	**0.01**
Cholesterol (mg/dL)	198.6 ± 22.1	204.3 ± 37.4	210.7 ± 49	184.6 ± 52.4	**0.009**	0.47	0.37	0.61	0.43	0.11	0.06
TG (mg/dL)	110.4 ± 48.1	137.2 ± 59.1	142.7 ± 69.3	130.1 ± 77.7	0.87	–	–	–	–	–	–
Creatinine (mg/dL)	0.85 ± 0.21	0.86 ± 0.18	1.55 ± 1.65	1.83 ± 1.65	**0.001**	0.26	**0.03**	**0.008**	**0.03**	**0.009**	0.38
GFR (mL/min/1.73m^2^)	94.4 ± 17.7	87.7 ± 16.5	70.8 ± 36.1	59.9 ± 34.5	**<0.0001**	0.31	**0.002**	**0.0002**	**0.01**	**0.0007**	0.12
Uric acid (mg/dl)	3.8 ± 1.01	5.7 ± 1.43	6.5 ± 2.17	6.2 ± 2.15	**<0.0001**	**0.007**	**0.001**	**0.002**	0.68	0.17	0.54
Potassium (mEq/L)	4.25 ± 0.37	4.34 ± 0.57	4.55 ± 0.87	4.47 ± 0.83	0.16	–	–	–	–	–	–
LogMMP-9	1.94 ± 0.3	1.92 ± 0.2	2.17 ± 0.3	2.31 ± 0.2	**< 0.0001**	0.60	0.05	**0.0003**	**0.001**	**0.0006**	0.94

Values are means ± standard deviation

*NT* normotensive group, *CHyp* controlled hypertensive group, *HypUrg* hypertensive urgency, *HypEmerg* hypertensive emergency, *HDL-c* high-density lipoprotein cholesterol, *LDL-c* low-density lipoprotein, *TG* Triglycerides, *GFR* glomerular filtration rate

There were significant differences in logMMP-9 levels between groups (*p*-value < 0.0001). The HypEmerg group (2.31 ± 0.29 ng/mL) had a significantly higher level of logMMP-9 compared to the NT (1.94 ± 0.32 ng/mL; *p*-value < 0.01) and CHyp groups (1.92 ± 0.23 ng/mL; *p*-value < 0.01). There were also significant differences between the HypUrg group (2.17 ± 0.3 ng/mL) and the NT (*p*-value < 0.05) and CHyp groups (*p*-value < 0.01). Although the logMMP-9 level was higher in the HypEmerg compared to the HypUrg group, no statistically significant difference was found.

The descriptive and ANOVA statistical analyses of the expressions of logMMP-9 are presented in Table 2. The correlation coefficients of logMMP-9 with clinical-biochemical variables that presented *p*-values < 0.05 in the univariate analysis were included for posterior multiple linear regression analysis, and the results are shown in Table 3. Age, diabetes history, SBP, DBP, glucose, creatinine, GFR and uric acid were evaluated to predict logMMP-9 and only uric acid was considered independent predictor for logMMP-9 increased.

**Table 3:** Multiple linear regression for log metalloproteinase-9

Variable	β	SEβ	CI (95%)	*P*-value
Age (years)	0.001	0.002	−0.002 to 0.005	0.763
SBP (mmHg)	0.001	0.001	− 0.001 to 0.004	0.286
DBP (mmHg)	−0.001	0.002	−0.005 to 0.003	0,674
Fasting glucose (mg/dL)	0.000	0.000	0.000 to 0.001	0.274
Serum creatinine (mg/dL)	−0.001	0.027	−0.054 to 0.052	0.982
GFR (mL/min/1.73m^2^)	0.001	0.001	−0.002 to 0.003	0.475
Uric acid (mg/dL)	0.041	0.012	0.019 to 0.064	**< 0.001**

*SBP* systolic blood pressure, *DBP* diastolic blood pressure, *GFR* glomerular filtration rate

There was no difference in logMMP-9 levels between the different clinical presentations of hypertensive emergencies (acute pulmonary edema, stroke, myocardial infarction, unstable angina and hypertensive encephalopathy; *P*-value = 0.9).

## Discussion

Matrix metalloproteinases-9 has been associated with several structural and functional changes of the cardiovascular system and, consequently, the development and progression of cardiovascular diseases [13, 14, 16–18]. In this study, MMP-9 expression was evaluated in hypertensive crisis, with levels that were progressively higher in the HypUrg and HypEmerg groups, suggesting that when inflammatory mechanisms are present [19, 20], the levels of MMP-9 may be associated with increased cardiovascular risk. These results also suggest that MMP-9 levels could constitute an important biomarker of cardiovascular risk, as well as the endothelial changes present in hypertension. Moreover, the mechanical disruption of the extracellular matrix of the arteries, caused by the mechanical stress of the BP or pulse pressure elevation itself, also participates in this process [21].

These data corroborate several clinical and experimental studies that described the association of MMP-9 levels with the incidence of acute cardiovascular disease and chronic hypertension [8–10, 13, 14, 16–18]. Hypertension is often associated with vascular remodeling and rearrangement of various components of the vascular wall including ECM. Several MMPs and tissue inhibitors of matrix metalloproteinase may be involved in the vascular remodeling associated with hypertension [22–24]. Increased MMP-9 activity could result in increased degradation of elastin relative to collagen leading to decreased elasticity [22, 23]. On the other hand, decreased endogenous tissue inhibitor of matrix metalloproteinase-1 (TIMP-1) activity could lead to accumulation of poorly cross-linked immature and unstable fibrin degradation products, resulting in misdirected deposition of collagen [22, 23]. Other studies also suggest that the increase of MMP-9 in healthy individuals may predispose them to cardiovascular diseases, because high levels of MMP-9 have been observed in acute cardiovascular events in individuals without known clinical diseases [17, 18].

In the current study, there was no significant difference in MMP-9 levels between the NT and CHyp groups, possibly due to the use of antihypertensive drugs by the hypertensive population. These data are corroborated by the presence of higher levels of MMP-9 in untreated hypertensive rats [24]. In addition, MMP-9 levels were reduced in clinical trials evaluating the effect of different antihypertensive drugs, such as calcium channel blockers (lercanidipine), while for others, such as felodipine, diltiazem and an angiotensin-converting enzyme inhibitor (enalapril), MMP-9 levels did not change, suggesting that the discrepancies in the results of different studies may result from drug therapy [5, 23, 25–28]. In this context, there is evidence that MMP-9 plays an important role in structural alterations associated with hypertension and its complications [24]. However, there are controversies regarding the expression of MMP-9 and its endogenous tissue inhibitor TIMP-1 reported in other studies indicating unchanged [9], higher [10, 23, 26, 27, 29] and even lower MMP levels [30, 31] to those of normotensive individuals. Therefore, it is important to consider the different drug classes used when assessing MMP-9 plasma levels.

The only independent explanatory variable for MMP-9 levels was uric acid (*p*-value = 0.002), as corroborated by studies that point to uric acid as an independent predictor for the development of hypertension [32, 33]. The association between elevated serum uric acid levels and markers of arterial stiffness, such as pulse wave velocity [34], corroborate our findings, in view of the action of MMP-9 in the degradation and reorganization of the vascular wall, and its consequent implication in the process of arterial stiffness [6, 7, 10, 14]. Moreover, uric acid contributes to systemic inflammation in humans and hypertension is an inflammatory disease [35, 36]. Thus, acute elevation of BP represents an inflammatory state more accentuated. MMP-9 is expressed constitutively at very low levels in bone marrow-derived cells; however, it is highly inducible under oxidative and inflammatory conditions [37]. Since in the hypertensive crisis, especially in the hypertensive emergency, inflammatory and coagulation pathways are activated [19, 20], it is plausible to find increased MMP-9 levels during the cases of acute increases of BP. Additionally, hyperuricemia is also a marker for reduced renal blood flow and plays a major role in the development and progression of kidney disease [38]. It is interesting to observe that our higher BP groups presented reduced GFR and the values for each group demonstrated the same degree of difference as those reported for MMP-9. Therefore, we cannot exclude that the increase in uric acid may be caused by a reduced GFR. However, on the other hand, some studies have suggested a role for MMPs and TIMPs in the progression of fibrosis in the kidneys (glomerular and tubulointerstitial), for both acute and chronic renal disease. In these cases, inflammatory mediators seem to be regulating MMP expression, and MMPs are proinflammatory mediators [39]. Thus, the reduced renal function, observed mainly in the cases of acute elevation of BP, impacts on the whole cardiovascular system through systemic modulations that may involve uric acid and MMP-9.

Despite the common occurrence of hypertensive crisis, the evidence regarding assessment of biomarkers in situations of acute hypertensive TOD is scarce. Studies have shown that asymptomatic individuals with markedly elevated BP without TOD do not benefit from acute reduction of BP [40, 41]. Thus, it is important to find a marker that can be used to increase diagnostic accuracy in this population, especially in the HypEmerg, or in those with suspicious clinical findings that require confirmation. Moreover, MMP-9 can be element representative of key biological pathways, pointing to a pathophysiologic mechanism involved in the TOD of the hypertensive emergencies, in which angiotensin II influences all the stages of the inflammatory response (vascular permeability, leukocyte recruitment and activation through selectins, integrins, adhesion molecules, cytokines and chemokines and vascular repair processes, including the participation of MMP-9) [42].

### Study strengths and limitations

We cannot fail to highlight the strength of the findings of this study that compared the MMP-9 levels in hypertensive crisis to normotensive and controlled hypertensive subjects, and also among different presentations of hypertensive emergencies, which has not been evaluated by other studies. Progressively, higher levels of MMP-9 were associated with higher BP levels present in cases of severe increases in blood pressure, defined as hypertensive crisis. However, some limitations need to be mentioned. First, it is a cross-sectional study, so the data do not allow the identification of cause-effect relationships. Secondly, the expression of TIMP-1, an inhibitor of MMP-9, was not evaluated, which could contribute to a better understanding of the relationship between MMP-9/TIMP-1 in hypertensive crisis. Finally, the analysis of MMP-9 levels was restricted to a single moment (during the hypertensive crisis). Ideally, the analysis of the MMP-9 in two moments, during the BP acute elevation and after the crisis, could provide information on the activation and inhibition of MMP-9 after the event, but the design of the study didn’t allow this evaluation. Nevertheless, the comparison between control (normotensive and controlled hypertensive individuals) and experimental (urgency and emergency hypertensive) groups showed significantly higher levels of MMP-9 during the acute elevation of BP.

## Conclusion

To the best of our knowledge, this is the first study that evaluates matrix metalloproteinase levels in hypertensive crisis, with MMP-9 levels being significantly higher in the hypertensive urgency and emergency groups compared to the other groups. Thus, these results suggest that the plasma MMP-9 levels may be associated with acute hypertensive events as participant of pathophysiologic pathways of the hypertensive crisis or a marker of acute elevation of blood pressure. In this context, new studies are needed to verify the modulation between the activation and inhibition of MMP-9, since its elucidation and control may constitute a diagnostic marker as well as a potential therapeutic agent.

## Data Availability

The datasets used and/or analyzed during the current study are available from the corresponding author on reasonable request.

## Abbreviations

ANOVA: Analysis of variance
BMI: Body mass index
BP: Blood pressure
CHyp: Controlled hypertensive group
CKD-EPI: Chronic kidney disease epidemiology collaboration
CVD: Cardiovascular diseases
DBP: Diastolic blood pressure
ECM: Extracellular matrix
GFR: Glomerular filtration rate
HDL-c: High-density lipoprotein cholesterol
HypEmerg: Hypertensive emergency group
HypUrg: Hypertensive urgency group
LDL-c: Low-density lipoprotein cholesterol
MMP: Matrix metalloproteinase
NT: Normotensive group
SBP: Systolic blood pressure
SD: Standard deviation
TC: Total cholesterol
TG: Triglycerides
TIMP-1: Tissue inhibitors of matrix metalloproteinase-1
TOD: Target organ damage
